# Computerized Quality of Life Assessment: A Randomized Experiment to Determine the Impact of Individualized Feedback on Assessment Experience

**DOI:** 10.2196/12212

**Published:** 2019-07-11

**Authors:** Daan Geerards, Andrea Pusic, Maarten Hoogbergen, René van der Hulst, Chris Sidey-Gibbons

**Affiliations:** 1 Patient-Reported Outcomes, Value & Experience Center Brigham and Women's Hospital Boston, MA United States; 2 Department of Surgery Harvard Medical School Boston, MA United States; 3 Department of Plastic and Reconstructive Surgery Catharina Hospital Eindhoven Netherlands; 4 Department of Plastic and Reconstructive Surgery Maastricht University Medical Center Maastricht Netherlands

**Keywords:** quality of life, outcome assessment, patient-reported outcome measures, computer-adaptive testing, WHOQOL, psychometrics, feedback

## Abstract

**Background:**

Quality of life (QoL) assessments, or patient-reported outcome measures (PROMs), are becoming increasingly important in health care and have been associated with improved decision making, higher satisfaction, and better outcomes of care. Some physicians and patients may find questionnaires too burdensome; however, this issue could be addressed by making use of computerized adaptive testing (CAT). In addition, making the questionnaire more interesting, for example by providing graphical and contextualized feedback, may further improve the experience of the users. However, little is known about how shorter assessments and feedback impact user experience.

**Objective:**

We conducted a controlled experiment to assess the impact of tailored multimodal feedback and CAT on user experience in QoL assessment using validated PROMs.

**Methods:**

We recruited a representative sample from the general population in the United Kingdom using the Oxford Prolific academic Web panel. Participants completed either a CAT version of the World Health Organization Quality of Life assessment (WHOQOL-CAT) or the fixed-length WHOQOL-BREF, an abbreviated version of the WHOQOL-100. We randomly assigned participants to conditions in which they would receive no feedback, graphical feedback only, or graphical and adaptive text-based feedback. Participants rated the assessment in terms of perceived acceptability, engagement, clarity, and accuracy.

**Results:**

We included 1386 participants in our analysis. Assessment experience was improved when graphical and tailored text-based feedback was provided along with PROMs (Δ=0.22, *P*<.001). Providing graphical feedback alone was weakly associated with improvement in overall experience (Δ=0.10, *P*=.006). Graphical and text-based feedback made the questionnaire more interesting, and users were more likely to report they would share the results with a physician or family member (Δ=0.17, *P*<.001, and Δ=0.17, *P*<.001, respectively). No difference was found in perceived accuracy of the graphical feedback scores of the WHOQOL-CAT and WHOQOL-BREF (Δ=0.06, *P*=.05). CAT (stopping rule [SE<0.45]) resulted in the administration of 25% fewer items than the fixed-length assessment, but it did not result in an improved user experience (*P*=.21).

**Conclusions:**

Using tailored text-based feedback to contextualize numeric scores maximized the acceptability of electronic QoL assessment. Improving user experience may increase response rates and reduce attrition in research and clinical use of PROMs. In this study, CAT administration was associated with a modest decrease in assessment length but did not improve user experience. Patient-perceived accuracy of feedback was equivalent when comparing CAT with fixed-length assessment. Fixed-length forms are already generally acceptable to respondents; however, CAT might have an advantage over longer questionnaires that would be considered burdensome. Further research is warranted to explore the relationship between assessment length, feedback, and response burden in diverse populations.

## Introduction

### Background

Quality of life (QoL) assessments conducted using questionnaires are an important feature of clinical research and are increasingly being used to inform clinical practice. They have allowed psychologists, epidemiologists, and health care researchers to accurately quantify aspects relating to a person’s QoL without relying on a structured interview with a trained professional. Though QoL questionnaires are commonly used in research studies and clinical trials, little research has been conducted to examine the effect of providing individualized feedback to people who complete these assessments, especially in QoL assessment [1,2].

In the context of health care provision, questionnaires that measure health and quality of life are often referred to as patient-reported outcome measures (PROMs). As an intervention designed to improve communication between patients and providers, PROMs can help health care providers understand what patients think about their own health. Gaining insight into patients’ own appraisal of their health is important, as research demonstrates that clinicians may have limited insight into the effects of illness on patients’ lives and cannot accurately predict how patients will rate their own mental and physical health [3,4]. PROMs are highly valued for their ability to address these problems [5-7].

Collection and feedback of PROMs in clinical practice can improve communication, decision making, satisfaction, and outcomes of care [8-11]. This information can be collected in many ways, ranging from basic paper-and-pen questionnaires to advanced computer systems. Research evidence suggests that only well-designed PROM interventions are likely to yield substantial improvements in clinical outcomes [12,13].

There are known barriers to using PROM questionnaires in both research and clinical practice. Doctors may avoid collecting PROMs because it can be difficult to relate to clinical decision making, and they fear it will add to clinical burden [5]. As patients or research participants, people may find questionnaires too burdensome or simply not interesting enough to justify completion [14].

### Burden

The burden of completing PROMs could be reduced by shortening assessments. Arbitrarily reducing the length of PROMs, however, would decrease the accuracy of the score estimates and the results. Computerized adaptive testing (CAT) is a technique that uses an algorithm to tailor questionnaire administration to individual patients and, as a result, is able to create short assessments while preserving accuracy [15,16]. Many simulation studies conducted in silico support the notion that CAT creates shorter assessments without sacrificing accuracy, and the assumption commonly presented is that shorter questionnaires always reduce burden, we are unaware of research that has evaluated the impact of shorter assessments on patient experience.

Along with reducing the length of questionnaires, the user experience of PROMs could be improved by making the assessment process more interesting and relevant. Advances in the power and availability of new computational tools indicate that CAT assessments can be readily deployed online alongside tailored graphical and text-based feedback. Research suggests that effective systems for collecting PROMs in clinical practice can be designed to capture information efficiently and provide clear feedback that makes it clear what should happen next; however, little is known about how these new technologies could be best used to improve user experience in this context [9,13,17].

### Objectives

In this study, we explore the impact of automatically generated personalized feedback on the user experience of electronic QoL assessment. We explore the following hypotheses:

- Providing immediate feedback to the respondent will increase acceptability and satisfaction of the assessment.
- Contextualizing QoL scores using tailored text-based feedback will improve user experience compared with graphical feedback only.
- Perceived accuracy of graphical feedback scores is the same in CAT as in fixed-length assessment.
- CAT improves user experience and is shorter than the fixed-length questionnaires.

## Methods

### Study Sample

**Figure 1:**
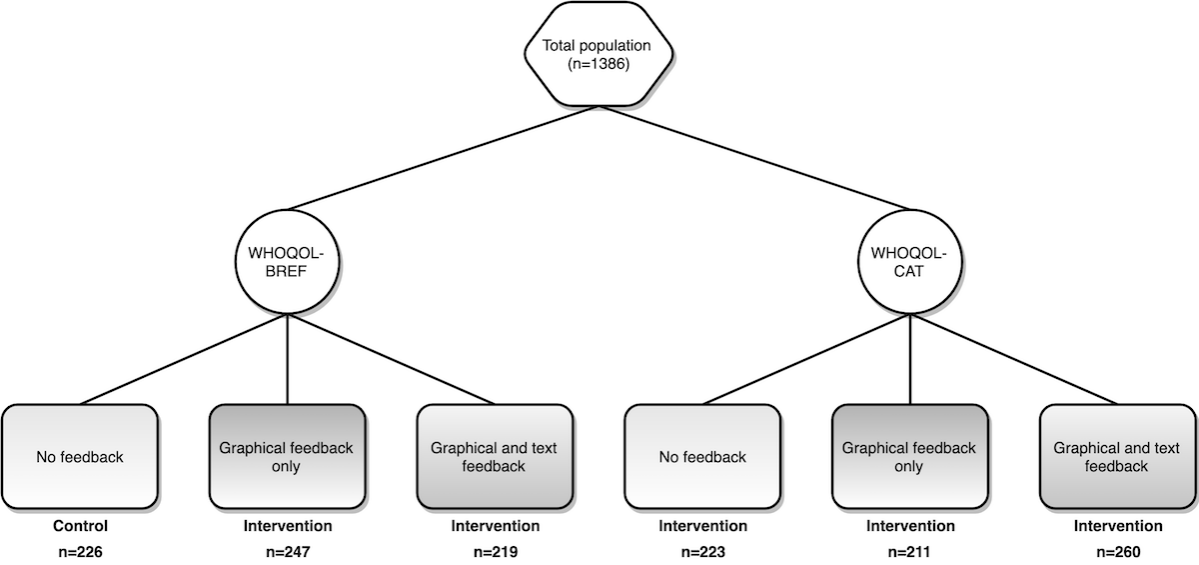
Experimental conditions. WHOQOL-BREF: abbreviated version of World Health Organization Quality of Life-100; WHOQOL-CAT: computerized adaptive test version of World Health Organization Quality of Life-100.

The sample consisted of participants from the general population in the United Kingdom. Participants were recruited between June 2017 and October 2017 through the Oxford Prolific Web panel, a crowdsourcing research platform [18]. We randomly assigned participants into 1 of 6 experimental conditions. In each condition, participants completed either a fixed-length QoL PROM or CAT QoL PROM [19-22]. In addition, they were randomly assigned to receive either no feedback, graphical feedback only, or graphical and adaptive tailored text-based feedback at the end of the questionnaire. Experimental conditions are displayed in Figure 1. Ethical approval was provided for this research by the institutional review board at the University of Cambridge Judge Business School (15-028).

### Measures

QoL assessments were based on the World Health Organization Quality of Life (WHOQOL) questionnaires [23-25]. The WHOQOL assesses different aspect of QoL, specifically physical health, psychological, social relationships, and environment. Responses are transformed to a score ranging from 0 (ie, worst QoL) to 100 (ie, best QoL). Each participant completed either a CAT version of the WHOQOL-100 (WHOQOL-CAT) or the fixed-length WHOQOL-BREF containing 24 items and 2 additional items assessing a respondent’s overall perception of their QoL and health [19-22].

The WHOQOL-BREF was scored in accordance with published guidelines, the WHOQOL-CAT was scored using a maximum likelihood estimation of theta scores using a single parameter *Rasch* partial credit item response theory method with *Z*-score transformation based on norm scores from the UK population [19,22]. The stopping rule of the WHOQOL-CAT was set at an SE below 0.45.

After the QoL assessment, participants completed a survey to assess engagement and acceptability. The feedback samples completed additional items regarding feedback accuracy and clarity. Responses were scored on a 5-point Likert scale (ie, disagree strongly, disagree a little, neither agree or disagree, agree a little, and agree strongly). In addition, we collected data on time spent viewing feedback. All experimental stimuli were derived using the Web-based open-source Concerto software (University of Cambridge Psychometrics Centre) [26]. To control for guessing and cheating, the item “I have not been paying attention,” was added, and respondents who endorsed this item were excluded from the analysis.

### Feedback

Graphical feedback was displayed as separate horizontal bar charts for each of the 4 WHOQOL domains, reflecting a score between 0 and 100. Text-based feedback included an explanation of what each domain reflects, how their score corresponds to average scores, and what their score might mean (eg, “Your score of 26 on this scale indicates that your psychological quality of life is lower than average. This suggests that your satisfaction with your psychological health is lower than it could be. You may be worrying more than usual, struggling to make decisions or not feeling content with your life. You could discuss these things with your doctor”). Feedback was augmented with a series of geographically relevant hyperlinks (assuming that the participant allowed their browser to access details of their location) to signpost relevant support services for each of the 4 domains. An example of how feedback was shown to participants can be seen in Multimedia Appendix 1.

### Analysis

All analyses were conducted within the R Statistical Programming Environment (version 3.4.4) [27]. Descriptive statistics were derived for age, gender, working status, and mean WHOQOL scores. We compared the research gold standard of a fixed-length QoL assessment without feedback (ie, control sample) with the other 5 conditions that are presented in Figure 1. The mean score and SD were derived for each survey item, including a summary score of all engagement and acceptability items (ie, total score of the 4 survey items). Separate item scores ranged from 0 to 4, where a score of 0 corresponded to the response “disagree strongly,” and a score of 4 corresponded to the response “agree strongly” (ie, 5-point Likert scale). The overall assessment score had a range of 0 to 16. Effect size for ordinal data was derived with Cliff delta (mean and range). Significance was assessed by performing Wilcoxon tests with a cutoff of *P*<.005 to increase reproducibility [28-31]. As proposed by Benjamin et al, a score of *P*<.05 was defined as suggestive instead of significant [30]. Time spent looking at feedback was compared between the *only graphical feedback* and the *graphical & text-based feedback* conditions.

We used the *mokken* package in R to perform Mokken scale analysis on the 4 acceptability and engagement items to assess unidimensionality and scalability. Scalability was displayed as Loevinger coefficient H, where a scale is considered weak if H<.3 and strong if H>.5. Unidimensionality was assessed by finding potential Mokken scales, with a cutoff set at .3 [32,33]. Furthermore, internal consistency of all 4 items were reflected with Cronbach alpha, derived by using the *psych* package in R [34]. A Cronbach alpha >.70 is generally seen as satisfactory when comparing groups [35,36].

## Results

### Study Sample

In total, 1454 participants completed the questionnaire. After excluding 68 respondents who endorsed the item “I have not been paying attention,” 1386 respondents were remaining for the analysis. Descriptive statistics (age, gender, working status, and mean WHOQOL scores) are presented in Table 1. The population distribution for all 6 conditions can be seen in Figure 1.

**Table 1:** Demographics (n=1386).

Characteristics		Statistics
**Age (years)**		
	Mean (SD)	40 (12)
	Range	18-75
**Gender, n (%)**		
	Female	669 (48.3)
	Male	544 (39.2)
	Not reported	173 (12.5)
**Working status, n (%)**		
	Full-time paid work (≥30 hours/week)	556 (40.1)
	Part-time paid work (<30 hours/week)	226 (16.3)
	Full-time education at school, college, or university	64 (4)
	Looking after home	134 (9)
	Fully retired from work	77 (5)
	Permanently sick or disabled	58 (4)
	Unemployed	55 (4)
**Mean World Health Organization Quality of Life scores, mean (SD)**		
	Physical	68 (18)
	Psychological	59 (17)
	Social	57 (17)
	Environmental	72 (13)

Scale and item properties Mokken analysis showed that all items were loaded on a single component, meaning that all items were assessing acceptability. Furthermore, Loevinger H was found to be .53 for the total scale, with all items having values >.30. Cronbach alpha was found to be .77.

### Overall Feedback and User Experience

When combining the WHOQOL-CAT and WHOQOL-BREF samples, providing graphical and tailored text-based feedback significantly improved overall experience compared with no feedback (mean_no feedback_ 11.2, SD 3.0; mean_graphical and text_ 12.3, SD 2.7; *P* ≤.001; Δ=0.22, Δ 95% CI 0.15-0.28). Providing only graphical feedback had a suggestive effect on overall user experience (mean_no feedback_ 11.2, SD 3.0; mean_graphical feedback_ 11.8, SD 2.9; *P*=.006; Δ=0.10, Δ 95% CI 0.02-0.17).

Text-based and graphical feedback was also found to improve user experience when comparing all samples separately. Furthermore, respondents thought the questionnaire with graphical and text-based feedback was more interesting compared with no feedback assessment, whereas providing only graphical feedback did not make the questionnaire more interesting. Participants who received graphical and text-based feedback were also more likely to report they would share the questionnaire with someone else. All results are presented in Table 2, in which every separate sample was compared with the control sample (fixed-length assessment without feedback).

**Table 2:** Assessment survey results. All samples are compared with the World Health Organization Quality of Life-BREF no feedback control sample.

Variable		World Health Organization Quality of Life-BREF			Computerized adaptive test version of World Health Organization Quality of Life-100		
		No feedback (n=226)	Graphical feedback (n=247)	Graphical & text-based feedback (n=219)	No feedback (n=223)	Graphical feedback (n=211)	Graphical & text-based feedback (n=260)
**Overall assessment (total of 4 items)**							
	Score (SD)	11.5 (2.88)	11.98 (2.74)	*12.31 (2.54)* ^b^	10.99 (3.09)	11.57 (3.13)	*12.35 (2.89)*
	Wilcoxon *P*	—^a^	.07	*.002*	.08	.72	*<.001*
	Δ^c^ (range)	—	0.09 (−0.01 to 0.19)	*0.16 (0.06 to 0.26)*	0.11 (−0.21 to 0.00)	0.01 (−0.09 to 0.12)	*0.18 (0.09 to 0.27)*
**“The questionnaire was interesting”**								
	Score (SD)	3.22 (0.79)	3.26 (0.81)	*3.43 (0.75)*	3.02 (0.92)	3.30 (0.81)	*3.46 (0.70)*
	Wilcoxon *P*	—	.41	*<.001*	.03	.17	*<.001*
	Δ (range)	—	0.04 (−0.05 to 0.13)	*0.17 (0.07 to 0.26)*	−0.11 (−0.21 to −0.01)	0.07 (−0.03 to 0.17)	*0.17 (0.08 to 0.26)*
**“I am satisfied with the amount of information”**								
	Score (SD)	3.29 (0.84)	3.35 (0.73)	3.44 (0.69)	3.19 (0.81)	3.30 (0.82)	3.37 (0.83)
	Wilcoxon *P*	—	.66	.07	.11	.99	.20
	Δ (range)	—	0.02 (−0.08 to 0.11)	0.08 (−0.01 to 0.18)	−0.08 (−0.18 to 0.01)	−0.01 (−0.10 to 0.09)	0.06 (−0.03 to 0.15)
**“It would be useful to share with someone else; perhaps my friends, spouse, or doctor”**								
	Score (SD)	2.12 (1.19)	2.43 (1.06)	*2.48 (1.04)*	2.11 (1.17)	2.24 (1.21)	*2.51 (1.08)*
	Wilcoxon *P*	—	.006	*.001*	.92	.32	*<.001*
	Δ (range)	—	0.14 (0.04 to 0.24)	*0.17 (0.07 to 0.27)*	−0.01 (−0.11 to 0.10)	0.05 (−0.05 to 0.16)	*0.18 (0.09 to 0.28)*
**“I would recommend this questionnaire to a friend”**								
	Score (SD)	2.90 (0.93)	2.94 (0.96)	2.97 (0.92)	2.69 (1.05)	2.76 (1.11)	2.98 (1.02)
	Wilcoxon *P*	—	.53	.38	.03	.35	.16
	Δ (range)	—	0.03 (-0.07 to 0.12)	0.04 (−0.06 to 0.14)	−0.12 (−0.22 to −0.02)	−0.05 (−0.15 to 0.05)	0.07 (−0.03 to 0.16)

^a^Contains no results since this was the control group for comparison with the other samples.

^b^Italicized results are significant (*P*<.005).

^c^Δ=Cliff delta.

No difference was found in perceived accuracy of the graphical feedback scores of the WHOQOL-CAT and WHOQOL-BREF (mean_CAT feedback accuracy_ 2.9, SD 0.9; mean_fixed feedback accuracy_ 3.1, SD 1.0; *P*=.05; Δ=0.06, Δ 95% CI 0.00-0.12). Furthermore, 757 out of 919 (82.4%) participants thought the graphical feedback was accurate, and 850 out of 915 (92.9%) participants thought the graphical feedback was clear. In the text-based feedback sample, 384 out of 469 (81.9%) participants affirmed accuracy of text-based feedback and 445 out of 468 (95.1%) affirmed clearness of text-based feedback. Response distribution of feedback appraisal is shown in Table 3.

**Table 3:** Feedback accuracy and clarity responses.

Feedback response	Responses, n	Disagree, n (%)	Neutral, n (%)	Agree, n (%)
The graphical feedback was accurate	919	83 (9.0%)	79 (8.6%)	757 (82.4%)
The graphical feedback was clear	915	27 (2.9%)	38 (4.2%)	850 (92.9%)
The text feedback was accurate	469	50 (10.7%)	35 (7.5%)	384 (81.9%)
The text feedback was clear	468	7 (1.5%)	16 (3.4%)	445 (95.1%)

### Computerized Adaptive Testing

CAT did not improve overall assessment experience scores compared with the fixed-length (mean_CAT_ 11.7, SD 3.1; mean_fixed_ 11.9, SD 2.7; *P*=.21; Δ=−0.06, Δ 95% CI −0.12 to 0.00). Even when combining adaptive assessment with graphical feedback, assessment experience did not significantly differ from the fixed-length assessment without feedback (mean_CAT_graphical_ 11.6, SD 3.1; mean_fixed_nofeedback_ 11.5, SD 2.9; *P*=.72; Δ=0.01, Δ 95% CI −0.09 to 0.12).

In the WHOQOL-CAT sample, mean items administered was 17.9 (SD 2.3), compared with 24 items in the WHOQOL-BREF, which corresponds to an item reduction of 25.4%.

### Feedback Time

Median time spent looking at feedback for all feedback groups combined was 129 seconds. Respondents in the WHOQOL-CAT sample spent significantly more time looking at graphical and text-based feedback compared with graphical feedback only, which is shown in Table 4. In the WHOQOL-BREF group, the difference in time looking at feedback did not comply to our *P* value threshold but has suggestive significance.

**Table 4:** Time spent looking at feedback. All samples are compared with the World Health Organization Quality of Life-BREF graphical feedback control sample.

Variable		World Health Organization Quality of Life-BREF		Computerized adaptive test version of World Health Organization Quality of Life-100	
		Graphical feedback (n=247)	Graphical & text-based feedback (n=219)	Graphical feedback (n=211)	Graphical & text-based feedback (n=260)
**Time spent looking at feedback**					
	Median, seconds	115	132	124	*147* ^a^
	Wilcoxon *P*	—^b^	.016	.42147	*<.001*
	Δ^c^ (range)	—	0.13 (0.02 to 0.23)	0.04 (−0.06 to 0.15)	*0.24 (0.15 to 0.34)*

^a^Italicized results are significant (*P*<.005).

^b^Contain no results since this was the control group for comparison with the other samples.

^c^Δ=Cliff delta.

## Discussion

### Conclusions

With this study, we have shown that immediately providing feedback after online QoL assessment significantly improves assessment experience when providing combined graphical and tailored text-based feedback. Graphical feedback alone did not improve assessment experience. Perceived accuracy of feedback was not different when comparing WHOQOL-BREF with WHOQOL-CAT, which suggests that CAT scores are as reliable as fixed-length scores, from a respondent’s perspective. The WHOQOL-CAT is shorter than fixed-length assessment, but it did not necessarily result in a better experience. Furthermore, respondents thought both graphical and text-based feedback after WHOQOL assessment were considerably clear and accurate.

### Other Literature

Little research has been conducted to assess QoL assessment feedback. Brundage et al assessed interpretation accuracy and ratings of ease of understanding and usefulness of different data presentation formats for both patients and respondents in both group-level data and individual-level data [1]. They looked at how graphical data should be provided and with which details, where we looked at, and what kind of feedback, including tailored text-based feedback, is most desired and found to be accurate by respondents. Kuijpers et al assessed self-rated understanding of Quality of Life Questionnaire-Core 30 scores and preference for presentation styles, whereas our research did not focus on presentation style but on feedback method [2].

### Strengths

This study has several strengths. The sample size is relatively large, with sufficient distribution in age, gender, and working status to accurately reflect the British population. The sample was large enough to establish 5 different large samples for comparison with our control group. By establishing 6 different samples, we were better able to target our different comparisons and hypotheses. We accepted a probability value of *P*=.005, rather than the conventional *P*=.05, to increase the likelihood of these results being replicable in future investigations. As the use of a *P* value of .005 might not be widely accepted yet, we have regarded a conventional *P* value of .05 as suggestive instead of significant [28-31].

We took steps to ensure our data were of high quality by adding dummy questions to the questionnaire to assess attentiveness and removing participants who stated that they were not paying attention, in line with recommendations for increasing reliability in studies conducted using compensated Web panels [37].

### Acceptability and Engagement Evaluation

In this study, we focused on the impact of feedback and impact of CAT on the user experience and assessed perceived accuracy of graphical feedback. As this represents 1 of the first efforts to do so, we were unable to find a previously validated questionnaire for assessing the relevance and acceptability of feedback and CAT administration. We developed a questionnaire that used items that had been shown to work well in the single other study we found that examined acceptability of feedback for patients completing a personality questionnaire [38]. Though we found that the questionnaire performed well during psychometric evaluation, we acknowledge that the short questionnaire may not cover all relevant aspects of questionnaire completion.

### Computerized Adaptive Testing

The questionnaire was, primarily, designed to assess experience relating to feedback (eg, the questionnaire was interesting; it would be useful to share with someone else, perhaps a friend, spouse, or doctor), which partially explains the lack of effect between the CAT and the fixed-length groups. In this study, the average for WHOQOL-CAT (SE<0.45) items administered was 17.9, which is only slightly more than expected based on an earlier simulation study conducted in silico (average 16.7, SE <0.45) [22]. This moderate item reduction in an already brief questionnaire might also explain why CAT did not affect user experience in this study. We know fixed-length questionnaires are likely to be acceptable to respondents, proven by their long-standing effective use. However, CAT is likely to provide an advantage over fixed-length forms that would be considered burdensome. Further research is therefore warranted to explore the relationship between CAT, questionnaire length, and patient burden.

### Assessment and Acceptability

Some unanticipated results were found. The scores on each questionnaire item were positively skewed in each group, indicating that although feedback did significantly improve experience, there appears to be something inherently positive about completing the WHOQOL questionnaire, regardless of the provision of feedback. Our experimental design prohibits us from understanding if the recruitment method (ie, via Web panel with compensation) affected the scores of acceptability measure, though participants were aware their reimbursement was not linked in any way to their responses. Studies that have been designed to assess the reliability of responses from similar Web panels (eg, Amazon’s Mechanical Turk) have found them to be reliable sources of information though, but to our knowledge, no studies have focused specifically at either QoL research or participants from the United Kingdom [39]. For evaluation of the assessment, we created our own survey items and conducted psychometric analyses to assess their suitability. Alternative for assessing feedback from clinical assessments are available, for example the “Patient Feedback Form” developed by Basch et al and adapted by Snyder et al, but unfortunately, this form has not been psychometrically validated for use in the English language. After we finished our data inclusion, Tolstrup et al translated and validated this evaluation form for a Danish patient population. Future research in this area may usefully validate the Patient Feedback Form for use in English [40-42].

### Impact

We discovered that the effect sizes were small to moderate. Despite the modesty of the effect sizes, we consider that adding tailored text-based feedback to outcome assessment might have a considerable impact on user experience, engagement, and response rate when feedback is implemented in outcome assessments where the primary goal is to maximize response rates and minimize longitudinal attrition. We chose a cross-sectional design to investigate this effect, and our positive results suggest that further experimentation in a cohort of patients who are prospectively followed up using QoL PROMs is warranted. In this study, we compared a participant’s scores to the population mean; during longitudinal assessment it becomes possible to feedback a person’s scores in relation to their previous scores, which may further increase the relevance of individualized feedback and therefore, the acceptability and willingness to participate in multiple PROM assessments over time.

### Bottom Line

In conclusion, providing feedback after outcome assessments is important to maximize user experience. Putting scores into context by using tailored text increased the user engagement. In addition, in this study, CAT did not improve overall experience but was substantially shorter than the fixed-length assessment. More research is necessary to assess CAT patient burden in terms of PROM assessment time, item reduction, patient-perceived length, and patient-perceived validity.

## Appendix

Multimedia Appendix 1 Example of feedback.

## Abbreviations

CAT: computerized adaptive testing
PROMs: patient-reported outcome measures
WHOQOL: World Health Organization Quality of Life
WHOQOL-BREF: abbreviated version of WHOQOL-100
WHOQOL-CAT: computerized adaptive test version of World Health Organization Quality of Life-100
