# Supplementary material for: Computerized Quality of Life Assessment: A Randomized Experiment to Determine the Impact of Individualized Feedback on Assessment Experience
Source: J Med Internet Res. 2019 Jul 11;21(7):e12212. doi: 10.2196/12212 (PMC6657452; doi:10.2196/12212)
Supplement: Multimedia Appendix 1 [file jmir_v21i7e12212_app1.pdf]

Personalised Quality of Life (QoL) Feedback

Scale	Score (out of 100)
Physical QoL	33
Psychological QoL	55
Social QoL	44
Environmental QoL	65

The scores above are worked out from the answers that you gave to the questions you have just completed. A higher score means that you have a higher quality of life.

More information on each of the domains is provided below.

Please read your feedback and then click the 'Next' button at the bottom of the page.

Physical Quality of Life

Your physical quality of life includes things like how well you are able to move around, how much energy you have or how much you are in pain.

Your score of 33 on this scale indicates that your physical quality of life is lower than average. This means that your satisfaction with your physical health is lower than it could be. This could mean that you are struggling with your mobility and doing the things that you enjoy. It may mean that you are in pain or finding it difficult to rest and sleep. Your doctor will be able to help you with some of these issues. We have provided some links to services below which may help you start to feel better.

If you are worried about your physical quality of life then you should contact your doctor. You can click here to find a local doctor or click here to access local emergency services.

Psychological Quality of Life

Your psychological quality of life includes things like how much confidence you have in yourself, negative feelings you experience or how much you value yourself.

Your score of 55 on this scale indicates that your psychological quality of life is normal. It looks like your psychological health is similar to most people in the United Kingdom. You can manage any negative feelings and often feel happy with your life. If this doesn't sound right, and you're feeling low then you could discuss these feelings with your doctor, who can help. You might also like to look at some of the links below.

If you have any concerns about you psychological quality of life you can click here to find help locally or click here to get in touch with the Samaritans.

Social Quality of Life

Social quality of life is all about your personal relationships and the support you get from your family and your friends.

Your score of 44 on this scale indicates that your social quality of life is normal. Your social quality of life is similar to most people in the United Kingdom. You feel well supported and engaged socially and have access to most of the information that you need. If this doesn't sound quite right, and you'd like some more help with your social quality of life you can click on the links below or ask your doctor.

For tips on things you could do to improve your social quality of life click here and for a NHS guide to social care and support click here.

Environmental Quality of Life

Environmental quality of life is all about how safe you feel in your home or work environment, the quality and availability of your transport and the opportunities you have for learning new information and skills.

Your score of 65 on this scale indicates that your environmental quality of life is very good. Your envrionmental quality of life is better than most people's in the United Kingdom. You have good access to transport, are generally satisfied with your financial situation and are happy with where you live. If this doesn't sound quite right, or you are interested to find out how you might improve your enviromental quality of life, or help someone else with theirs, then have a look at the links below.

If you have any issues with your environmental quality of life then you could contact your local council or click here to access local emergency services.
